# Salivary diagnostics: Bridging dentistry and medicine: A systematic review

**DOI:** 10.6026/9732063002001754

**Published:** 2024-12-31

**Authors:** Deepa Benni, Pallavi K.M, Tejaswi Maddukuri, Richa Singh, Jayant Kumar Gahlot, Parama Basu, Anukriti Kumari

**Affiliations:** 1 Department of Dentistry, Karnataka Medical College and Research Institute, Hubballi, India; 2 Department of Dentistry, School of Dental Sciences, Sharda University, Greater Noida, Uttar Pradesh, India; 3 Department of Dentistry, Lenora institute of dental science, NTR University, Rajanagaram, Rajamahendravaram, Andhra Pradesh, India; 4 Department of Oral and Maxillofacial Surgery, Shree Bankey Bihari Dental College & Research Centre, Ghaziabad, India; 5 Department of Oral Medicine and Radiology, Santosh Dental College, Ghaziabad, Uttar Pradesh, India; 6 Department of Periodontics, House Surgeon, Dr. R Ahmed Dental College and Hospital, Kolkata, West Bengal, India; 7 Department of Oral Medicine and Radiology, School of Dental Sciences, Sharda University, Greater Noida, India

**Keywords:** Biomarkers, diagnostic, saliva

## Abstract

Saliva has emerged as a non-invasive, easily accessible biofluid for diagnostic purposes in dentistry and medicine. Biomarkers in saliva, such as proteins, enzymes, nucleic acids and metabolites, reflect both systemic and oral health. Advances in molecular science, nanotechnology and bioinformatics have enabled highly accurate assays for early disease detection, monitoring and treatment guidance. Salivary diagnostics are promising for identifying conditions like diabetes, HIV, COVID-19, autoimmune disorders, cardiovascular diseases and oral diseases such as periodontal disease and oral cancer. To fully realize its potential, regulatory approval, standardized procedures and further research on salivary biomarkers are essential.

## Background:

Salivary biomarkers are biologically active substances derived from saliva which can reflect both oral and systemic health. They are categorized into various categories based on their molecular nature and the types of ailments that they help diagnose [1]. A broad range of molecules make up salivary biomarkers. The immune response, inflammation and microbial defence are some of the functions that are highly reliant on proteins and peptides, comprising enzymes (like lacto-ferrin, amylase), immune-globulins (like IgA) and cytokines (like IL-1β, TNF-α). IgA is essential for mucosal immunity, but cytokines like TNF-α and IL-1β play a key role in the inflammatory response seen in periodontal disease. Enzyme-linked immunosorbent assays (ELISA), Western blotting and proteomics approaches like mass spectrometry (MS) are often employed to isolate these substances [2]. Crucial salivary biomarkers include nucleic acids, like messenger RNA (mRNA), microRNA (miRNA) and genomic DNA. Though salivary DNA can be used in epigenetic analysis, mutation detection and genetic screening, mRNA and miRNA are used as markers of active gene expression in diseases like viral infections, cancer and cardiovascular disease [3]. After isolating the DNA or RNA out of saliva, these nucleic acids are separated by PCR-based techniques like next-generation sequencing (NGS), reverse transcription-PCR (RT-PCR) and quantitative PCR (qPCR). Biomarkers for physiological states and metabolic activity include lactate, cortisol, glucose and urea. While cortisol levels are used for stress response and adrenal function, elevated salivary glucose levels can indicate inadequate glucose control in diabetes. Methods like gas chromatography (GC), liquid chromatography-mass spectrometry (LC-MS), or nuclear magnetic resonance (NMR) spectroscopy serve to recognize and measure metabolites [4].

Membrane integrity and signalling constitute two physiological processes that are impacted by salivary lipids, including fatty acids, cholesterol and lipid peroxidation products. Atherosclerosis, diabetes and oral malignancies are among the disorders that alter lipid levels. Solid-phase extraction, or SPE, is often used for separating lipids and chromatography or mass spectrometry is employed for analysis. The body's general electrolyte homeostasis and oral pH equilibrium rely on minerals and electrolytes such sodium, potassium, calcium, phosphate and chloride. Variations in potassium and sodium levels could mean renal failure or dehydration [5]. Utilizing flame photometry, atomic absorption spectrophotometry and ion-selective electrodes, these ions are gauged. Salivary hormones, such as insulin, testosterone, cortisol and estradiol, represent systemic endocrine activity. Stress is mostly indicated by cortisol and reproductive health appears by testosterone and estradiol [6]. ELISA and radioimmunoassay (RIA) are two basic immunoassays employed to isolate hormones. Last but not least, pathogens like Porphyromonas gingivalis (linked to periodontitis) or viral RNA from SARS-CoV-2 (COVID-19) are recognized via microbial components such bacterial DNA, viral RNA and fungal antigens. To aid in early infectious disease detection & monitoring, these microbial factors are found by means of PCR, next-generation sequencing (NGS), or microbial culture techniques [7].

## Methodology:

## Study design:

**Figure 1:**
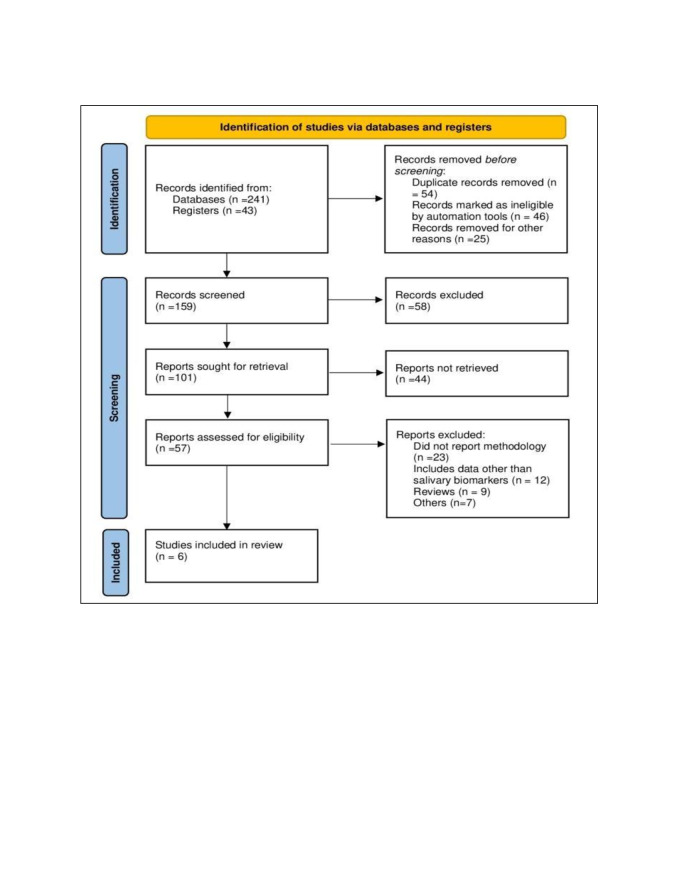
Prisma flowchart of the study

The purpose of this study was to assess the clinical value of salivary biomarkers for the diagnosis of systemic and oral disorders. It was planned as a systematic review. The study concentrated on identifying different biomolecules found in saliva, their potential as diagnostic tools and their applicability to the dentistry and medical domains. The study followed Preferred Reporting Items for Systematic Reviews and Meta-Analyses (PRISMA) criteria (Figure 1).

## Strategy:

A thorough search of the literature was done using a number of internet databases, including Google Scholar, PubMed, Scopus and Web of Science. To find pertinent research published between January 2010 and August 2024, a search was conducted. Salivary biomarkers, oral illnesses, systemic disorders, diagnosis, proteomics, genomics and periodontal diseases were among the search phrases used. To hone the search, boolean operators ("AND", "OR") were employed. A manual search of the reference lists of pertinent papers was also conducted to find other research.

## Study selection:

The following were the review's inclusion criteria:

Studies of the following types include cross-sectional studies, cohort studies, case-control studies, randomised controlled trials (RCTs) and systematic reviews.

## Participants:

Human patients identified using salivary biomarkers as having systemic illnesses, oral disorders, or both. Research assessing salivary biomarkers' clinical significance, sensitivity, specificity and accuracy as diagnostic tools was completed. Studies were excluded if they lacked quantitative data, were conducted on animal models, or did not focus specifically on salivary biomarkers.

## Data extraction and quality assessment:

Two reviewers separately carried out the data extraction. The study's title, author(s), year of publication, journal, goal, methodology, kind of biomarker, techniques for diagnosis and results were all retrieved. The Newcastle-Ottawa Scale (NOS) for cohort studies and the Cochrane Risk of Bias tool for RCTs were used to evaluate the quality of the chosen studies. Inconsistencies in the extraction of data or the evaluation of quality were settled by consensus-building among reviewers.

## Biomarker analysis:

## Techniques:

The studies that analysed salivary biomarkers included cytokines, microRNAs, proteins, metabolites, hormones and nucleic acids. Various analytical methods were used in each study, such as next-generation sequencing (NGS), mass spectrometry (MS), polymerase chain reaction (PCR) and enzyme-linked immunosorbent assays (ELISA). The molecular properties of the biomarkers under investigation determined the technique of choosing.

## Statistical analysis:

The results were summarized using descriptive statistics and forest plots were created to show the diagnostic odds ratios (DORs) and pooled sensitivity and specificity for certain salivary biomarkers. The I2 statistic was used to measure the heterogeneity between the studies; values more than 50% indicated significant heterogeneity. Subgroup analyses were carried out where needed to investigate possible sources of heterogeneity. To take study variability into account, a random-effects model was used. Review Manager (RevMan) version 5.4 was used for all statistical analyses.

## Review:

The domain of salivary diagnostics continues to grow swiftly, taking advantage of saliva's non-invasive capabilities as a biofluid to monitor and cure systemic and oral disorders. A broad spectrum of biomarkers, comprising hormones, microbial DNA, microRNAs, cytokines and enzymes, can be identified in saliva and suggest pathological alterations in both the mouth cavity and other distant organ systems [8]. Salivary biomarkers have demonstrated enormous promise in dentistry for improving accuracy in diagnostics and establishing individualized strategies for treatment in early detection of periodontal conditions, dental caries and oral cancer. Furthermore, salivary diagnostics can be used to identify systemic diseases like diabetes mellitus, cardiovascular disease, autoimmune disorders (like Sjögren's syndrome) and viral infections (like HIV, COVID-19) [9]. Biomarkers like glucose, C-reactive protein (CRP) and viral RNA may offer substantial information into the advancement of these ailments. Owing to their frequent patient interactions, dentists play a vital role in establishing collaborative relationships with healthcare providers and implementing salivary diagnostics into practice for the prompt identification of disease [10]. The clinical importance of salivary diagnostics in screening, monitoring of diseases and preventive medicine is growing due to the non-invasive nature of saliva collection and the increasing availability of molecular assays like PCR and ELISA. Salivary diagnostics, offering a useful, patient-friendly approach to health-related management, has the potential to completely transform dental and medical practice as biomarker identification and diagnostic technology continue to advance [11] Table 1 summarizes studies exploring salivary biomarkers' diagnostic roles, focusing on systemic and oral diseases.

## Discussion:

Compared to blood draws or tissue biopsies, saliva collection is an easy, painless and non-invasive technique. Specially for long-term monitoring of oral and systemic disorders, this method minimises patient discomfort and improves compliance. Salivary diagnostics are also a cost-effective choice with little training and equipment, making them appropriate for community and clinical settings along with large-scale screening [12]. The capacity to identify molecular alterations in the absence of clinical signs allows for the early detection of diseases as oral cancer, periodontal disease and systemic ailments including diabetes and cardiovascular disorders. High levels of salivary matrix metalloproteinases (MMPs) may serve as an indicator of the early stages of periodontal tissue damage. With biomarkers like IL-1β, IL-6 and TNF-α helpful in determining the inflammatory status in oral disorders, the dynamic nature of saliva permits immediate tracking of progressing illness and therapy responses [13]. As an instance, oral squamous cell cancer (OSCC) is markedly linked with an overexpression of specific salivary miRNAs, such as miR-125a and miR-31 [14]. Salivary biomarkers also serve as indicators of systemic health; for example, high cortisol levels indicate systemic stress or adrenal malfunction, whilst excessive glucose levels imply hyperglycaemia in diabetic individuals [15].

For point-of-care applications, saliva-based diagnostics are perfect because they provide quick answers that let clinicians make decisions quickly. In dental settings, tests like salivary enzyme-linked immunosorbent assays (ELISA) might be employed to evaluate biomarkers like MMP-8 or C-reactive protein (CRP), providing quick feedback [16]. Furthermore, saliva is a non-infectious biofluid that reduces the threat of blood-borne pathogen contamination and dissemination, benefiting both patient and physician safety [17]. By defining distinct molecular profiles that inform precision treatments-particularly for chronic periodontitis-salivary biomarkers also aid in customised medicine. Biomarkers such as IL-6 and IL-8 could provide constant insight into inflammation and disease status in time, making saliva collection especially helpful for longitudinal analysis of chronic conditions like diabetes and periodontal disease [18].

For geriatric and paediatric populations, where surgery may be challenging, saliva collection is especially appropriate due to its non-invasive nature. Salivary diagnostics helps detect oral infections and control the administration of antibiotics by detecting microbiological DNA or RNA. Saliva is also useful in tracking hormones and stress; cortisol, for example, helps identify Cushing's syndrome, Addison's disease and stress levels prior to and after dental procedures. Finally, because saliva only needs a tiny sample volume, people who have decreased saliva flow due to ageing, medicine, or systemic disorders nevertheless use saliva as a screening alternative [19]. Functions of Individual Biomarkers have been described in Table 2.

Utilising non-invasive diagnostic and monitoring tools for early detection as well as personalised therapy, salivary biomarkers find extensive use in multiple disciplines of dentistry. To assess the extent of periodontal swelling and tissue loss in conditions like chronic periodontitis, periodontologists utilise biomarkers such as Matrix Metalloproteinase-8 (MMP-8) and Tumour Necrosis Factor-alpha (TNF-α) [20]. Clinicians may apply these markers to monitor the immune system response of the host and advise therapies like applying antibiotics or scaling and root planning. MicroRNAs (miR-21, miR-31) and cytokines (IL-8) are useful biomarkers in oral and maxillofacial pathology for early identification of oral squamous cell carcinoma (OSCC), which boosts prognosis through facilitating prompt diagnosis, especially for high-risk groups such as smokers or those who were exposed to the human papillomavirus (HPV) [21]. Notably in the cases of apical periodontitis and pulp necrosis, biomarkers like Interleukin-6 (IL-6) and Interleukin-10 (IL-10) might be employed in endodontics to direct endodontic therapy and evaluate inflammation that occurs in pulpal infections [22]. Analogously, salivary levels of osteocalcin and C-terminal telopeptide of type I collagen (CTX-1) are analysed in orthodontics to assess how the bone changes when teeth move and to maximise the effectiveness of treatment [23]. In prosthodontics, biomarkers such as lactate dehydrogenase (LDH) and creatine kinase (CK) are explored to assess the health of oral mucosa under prosthetic appliances like dentures, helping to detect early mucosal damage or trauma. For dental caries risk assessment in paediatric dentistry, salivary levels of Streptococcus mutans and Lactobacillus species are crucial in predicting caries susceptibility, enabling preventive interventions like fluoride application or sealants [24].

The detection of autoimmune diseases like Sjögren's syndrome is made feasible in oral medicine by salivary diagnostics, providing a non-invasive way of keeping track of and diagnosing autoimmune illnesses by the detection of Anti-Ro/SSA and Anti-La/SSB antibodies [25]. In geriatric dentistry, biomarkers such as osteopontin and C-reactive protein (CRP) are additionally employed to track the progression of systemic illnesses like osteoporosis and other age-related disorders affecting dental health. Multiple applications of salivary biomarkers illustrate how crucial they are to the progression of individualized dental care in an assortment of specializations. Salivary biomarkers have a wide range of uses in medicine. Salivary leptin and insulin levels serve a purpose in monitoring insulin resistance and metabolic syndrome, while salivary cortisol is often employed in endocrinology to assess adrenal function and aid in making diagnoses of Cushing's syndrome and Addison's disease. In the field of diabetology, poorly controlled diabetes mellitus is detected by higher salivary glucose and inflammatory markers such as Interleukin-6 (IL-6) and Tumor Necrosis Factor-alpha (TNF-α), which offer valuable insights into the control of metabolism and systemic inflammation [26].

Salivary C-reactive protein (CRP), myeloperoxidase (MPO) and lipoprotein-associated phospholipase A2 (Lp-PLA2) are markers of systemic inflammation in cardiology. They are associated with the risk of atherosclerotic cardiovascular disease (CVD) and can be used to help identify and stratify patients based on their risk for conditions like stroke and myocardial infarction early on. Salivary microRNAs (miR-21, miR-200) and p53 antibodies are used in oncology to identify tumours non-invasively, including pancreatic, breast and oral squamous cell carcinoma (OSCC), which improves prognosis by detecting the disease early [27]. In immunology, salivary TNF receptors and Interleukin-8 (IL-8) are used to track inflammatory responses in conditions like rheumatoid arthritis and systemic lupus erythematous (SLE). Salivary Anti-Ro/SSA and Anti-La/SSB antibodies are essential for the diagnosis of Sjogren's syndrome, an autoimmune disease that affects exocrine glands [28].

In psychiatry and neurology, salivary cortisol and alpha-amylase are employed to evaluate the hypothalamic-pituitary-adrenal (HPA) axis and autonomic nervous system (ANS) function, which are crucial for understanding stress-related disorders such as depression, anxiety and post-traumatic stress disorder (PTSD). Furthermore, in neurodegenerative diseases, salivary tau protein and beta-amyloid levels are being investigated as potential biomarkers for Alzheimer's disease, allowing for early detection and progression monitoring [29]. Large-scale, non-invasive methods for screening have been rendered feasible by salivary immunoglobulin A (IgA) and cytokines that promote inflammation, which serve as early indicators of viral infections like COVID-19 and HIV in the care of infectious diseases. These diverse uses highlight the growing contribution that salivary biomarkers have contributed in customizing treatment and enhancing patient outcomes in many different kinds among healthcare professions [30].

## Conclusion:

Salivary biomarkers offer valuable insights into both dental and systemic health, aiding in early detection and personalized care. Advances in molecular techniques are enhancing the ability to monitor diseases in real-time and improve patient outcomes through personalized medicine. Integrating salivary diagnostics into dentistry allows for comprehensive health management, enabling early detection, treatment and monitoring of various systemic and oral conditions.

## Figures and Tables

**Table 1:** Review of the recent literature

Authors	Year	Title	Journal	Objective	Methodology
Roi A *et al.* [14]	2019	A New Approach for the Diagnosis of Systemic and Oral Diseases Based on Salivary Biomolecules	Dis Markers	To explore the role of salivary biomolecules in diagnosing systemic and oral diseases	Review on salivary biomolecules and their diagnostic potential.
Javaid MA *et al.* [16]	2016	Saliva as a diagnostic tool for oral and systemic diseases	J Oral Biol Craniofac Res	To highlight the diagnostic capabilities of saliva for both oral and systemic diseases.	Review of studies on saliva as a non-invasive diagnostic tool.
Meleti M *et al.* [11]	2020	Salivary biomarkers for diagnosis of systemic diseases and malignant tumors. A systematic review	Med Oral Patol Oral Cir Bucal	To assess the effectiveness of salivary biomarkers in diagnosing systemic diseases and malignancies.	Systematic review of studies on salivary biomarkers.
Nagler RM *et al.* [4]	2023	Salivary Biomarkers for Oral and Systemic Diseases	Frontiers in Dental Medicine [17]	To evaluate salivary biomarker potential in diagnosing both oral and systemic conditions like cancer and Covid-19	Analysis of saliva components (proteins, RNA) through omic approaches
Hu, Hongying *et al.* [12]	2023	Mass Spectrometry-Based Proteomics for Discovering Salivary Biomarkers in Periodontitis	International Journal of Molecular Sciences	Review the potential of mass spectrometry to identify salivary biomarkers for periodontitis	Systematic review of proteomic studies using mass spectrometry on saliva samples
Korte DL *et al.* [6]	2016	Personalised Medicine: An Update of Salivary Biomarkers for Periodontal Diseases	Periodontology 2000	Explore personalised medicine using salivary biomarkers for periodontal disease detection	Systematic review on the role of specific biomarkers in diagnosing periodontal diseases

**Table 2:** Functions of individual biomarkers

Biomarker	Function	Associated conditions
Cytokines (*e.g.,* IL-6, IL-8, TNF-α)	Serve as inflammatory markers. Elevated levels indicate inflammation.	Periodontitis, oral cancers, systemic inflammatory conditions
Cortisol	A marker for psychological and physical stress, as well as adrenal gland function.	Stress, Cushing's syndrome, depression
microRNA (*e.g.,* miR-31, miR-21)	Small RNA molecules that regulate gene expression and serve as disease biomarkers.	Cancer, cardiovascular diseases, neurological disorders
Amylase	Enzymes responsible for carbohydrate breakdown, elevated during stress.	Sympathetic nervous system activity, psychological stress
Glucose	Increased levels indicate diabetes and correlate with blood glucose.	Diabetes mellitus
